# OSCC Exosomes Regulate miR-210-3p Targeting EFNA3 to Promote Oral Cancer Angiogenesis through the PI3K/AKT Pathway

**DOI:** 10.1155/2020/2125656

**Published:** 2020-07-08

**Authors:** Hui Wang, Lin Wang, Xiaocheng Zhou, Xinyue Luo, Ke Liu, Erhui Jiang, Yang Chen, Zhe Shao, Zhengjun Shang

**Affiliations:** The State Key Laboratory Breeding Base of Basic Science of Stomatology (Hubei-MOST) & Key Laboratory of Oral Biomedicine Ministry of Education, School & Hospital of Stomatology, Wuhan University, China

## Abstract

This study is aimed at determining how oral squamous cell carcinoma (OSCC) regulates the angiogenesis of HUVECs through miR-210-3p expression and exploring the relationship among miR-210-3p, its target protein, and the possible mechanism of angiogenesis regulation. miR-210-3p expression was detected in OSCC tissues and juxta cancerous tissues (JCT), and the relationship among miR-210-3p, microvessel density (MVD), and histopathologic features was analyzed. A conditioned medium (CM) of the OSCC cell line CAL27 was collected to stimulate human umbilical vein endothelial cells (HUVECs), and the miR-210-3p levels and tube formation capability of HUVECs were measured. The target protein level of miR-210-3p was altered; then, PI3K/AKT pathway activation in HUVECs was detected. miR-210-3p was tested in exosomes separated from CAL27 CM, and the transfer of miR-210-3p from OSCC exosomes to HUVECs was verified. Then, we found that the OSCC tissues had higher miR-210-3p levels than the JCT, and miR-210-3p level was positively correlated with MVD and tumor grade. CAL27 CM was able to elevate miR-210-3p levels in HUVECs and promoted tube formation. EFNA3 was the target gene of miR-210-3p, and ephrinA3 protein level was able to influence the migration and proliferation of HUVECs. The levels of phosphorylated AKT in the HUVECs increased when ephrinA3 was downregulated, and the upregulation of ephrinA3 resulted in the suppression of the PI3K/AKT pathway. miR-210-3p was detected in exosomes isolated from the CM of CAL27 cells, and miR-210-3p level in the HUVECs was elevated after absorbing the OSCC exosomes. In conclusion, miR-210-3p was more overexpressed in OSCC tissues than in the JCT. The exosomes secreted by OSCC cells were able to upregulate miR-210-3p expression and reduce ephrinA3 expression in HUVECs and promoted tube formation through the PI3K/AKT signaling pathway.

## 1. Introduction

Oral cancer is a general designation of all malignant tumors appearing in the mouth. In 2018, the number of new oral cancer cases was approximately 354,000, accounting for 2% of the total number of 36 cancer cases. Oral squamous cell carcinoma (OSCC) cases constitute 90% of all oral carcinoma cases [1]. Given the poor survival rates and living quality of patients with OSCC, exploring the molecular mechanisms of OSCC progression is urgently necessary for identifying biomarkers or targeted sites for treatment.

Tumors cannot grow without absorbing nutrients and oxygen and eliminating cellular waste, and these processes are mostly dependent on the construction of the vasculature [2]. Thus, tumor angiogenesis is a crucial and complicated event during tumor progression [3]. The distribution of tumor vessels is tightly associated with tumor stage, risk of metastasis, and overall survival in many kinds of tumors [4, 5]. At present, antiangiogenic therapy is considered an effective strategy for fighting tumors [6, 7]. Furthermore, employing antiangiogenic medicines and proteins in animal models and in vitro experiments can markedly suppress tumor development; therefore, angiogenesis is a valid target for OSCC treatment [8–10].

MicroRNAs play critical roles in the regulation of target gene expression after transcription by engaging in complementary base pairing with the 3′- untranslated region (UTR) of a target mRNA; they constitute a class of single-stranded small-molecule noncoding RNAs, which contain approximately 22 nucleotides [11–14]. Accumulated evidence has shown that microRNAs participate in tumorigenesis and promote tumor proliferation, migration, and angiogenesis and epithelial-mesenchymal transition (EMT) [15, 16]. Recent studies have indicated that the overexpression of miR-210-3p can facilitate tube formation and migration in HUVECs [17]. However, the exact mechanism of miR-210-3p in OSCC progression and the signal pathway involved remains indistinct.

Ephrin is the ligand of the Eph receptor. EphAs preferentially combine with EphrinAs with five glycosylphosphatidylinositols; meanwhile, EphBs have high-affinity binding domains with three transmembrane ephrinB (ephrinB1-B3) receptors [18, 19]. Ephrins play important roles in normal physiological processes, such as embryo organisational boundary formation and axon development, and facilitate wound healing in adult tissues. In addition, these receptors and ligands are abnormally expressed in some pathological processes, such as tumor and neurodegenerative disorders [20], and participate in angiogenesis in vivo and in vitro [21]. EphrinA3 can participate in angiogenesis, but its specific mechanism is still unclear [17, 22, 23]. Several microRNAs target the Eph/ephrin family and regulate some biological function [24, 25].

As a critical mediator of information exchange between cancer cells and the surrounding mesenchyme in tumor microenvironments (TMEs), exosomes released by cells enclose small vesicles, messenger RNAs, microRNAs, and bioactive proteins and deliver them to recipient cells [26, 27]. Exosomes secreted by many kinds of tumors promote tumor angiogenesis by delivering functional microRNAs and proteins to stroma cells in the TME [28, 29]. This course, along with other effects, such as an increased rate of extracellular matrix degradation and remodelling, accelerates tumor invasion into the stromata, thereby expediting tumor development. After being absorbed by recipient cells, microRNAs in exosomes modulate the expression of target genes and exert an influence on biological behaviour in the recipient cells [30–32].

The present study was aimed at clarifying the influence of miR-210-3p on OSCC exosomes on endothelial cells in a TME and exploring the potential underlying mechanisms.

## 2. Materials and Methods

### 2.1. Cell Line and Culture

Human umbilical vein endothelial cells (HUVECs) were purchased from ScienCell (Carlsbad, CA, USA) and cultured with ECM (ScienCell, Carlsbad, CA, USA) containing 5% FBS (ScienCell, Carlsbad, CA, USA). The OSCC cell line CAL27 was purchased from the China Center for Type Culture Collection (Shanghai, China) and cultured with high-glucose DMEM (HyClone, UT, USA) containing 10% FBS (Natocor, Córdoba, Argentina). Both of them are cultured in a humidified incubator containing 5% CO_2_ at 37°C. Short tandem repeat was performed routinely on these cell lines to confirm their authenticity, and *Mycoplasma* was routinely tested.

### 2.2. Patients and Tumor Tissues

Human OSCC tumors (*n* = 80) and normal mucosa tissues (*n* = 7) were collected at Hospital of Stomatology, Wuhan University, between 2017 and 2018. None of the patients in our study received chemotherapy or radiotherapy. Informed consent was obtained from all patients, and approval from the Ethics Committee of Hospital of Stomatology, Wuhan University, was obtained to perform histological analysis of the samples above.

### 2.3. RNA Extraction, Reverse Transcription, and Real-Time PCR

Total RNA was extracted from cells and exosomes using the TRIzol reagent (Takara, Tokyo, Japan). cDNA was transcribed from 400 ng of RNA using the PrimeScript Reverse Transcription Reagent Kit (Takara, Tokyo, Japan). Real-time quantitative PCR was then performed with SYBR® Premix Ex Taq™ II (Takara, Tokyo, Japan). RNA was reverse-transcribed using miRNA first-strand cDNA synthesis (Sangon Biotech, Shanghai, China) and then quantified by using the MicroRNA qPCR Kit in triplicate (SYBR Green method) (Sangon Biotech, Shanghai, China). All processes were performed following the manufacturer's instructions. U6 was used as the internal reference for miR-210-3p, while glyceraldehyde-3-phosphate dehydrogenase (GAPDH) was used as the internal reference for other genes. A universal reverse primer was provided by Sangon Biotech (no. b661601). QPCR was conducted on QuantStudio™ 6 Flex (Life Technologies, USA). RNA and miRNA primers synthesized by Sangon Biotech (Shanghai, China) are shown in Table 1.

### 2.4. Western Blotting Analysis

The total protein of cells and exosomes was extracted by using a mammalian protein extraction reagent (MPER) (Thermo Fisher Scientific) with protease and phosphatase inhibitors on ice. The protein concentration of every sample was measured by using a bicinchoninic acid protein assay kit (Thermo Fisher Scientific Inc., USA). Then, the loading buffer (5×) was mixed with protein solutions and heated for 5 min at 95°C. 20 *μ*g of protein was added to 10-12% SDS-PAGE for 30 min at 60 V and then for 70 min at 110 V. Next, the proteins were transferred to a polyvinylidene difluoride (PVDF) membrane in cold transfer buffer for 90-120 min at 200 mA. After blocking with 5% skim milk in Tris-buffered saline containing 0.05% Tween 20 (TBST) at room temperature for 2 h, the membranes were incubated with antibodies at 4°C overnight. Antibodies used in experiments are as follows: glyceraldehyde-3-phosphate dehydrogenase (GAPDH; Proteintech, Wuhan, China), ephrinA3 (Proteintech, Wuhan, China), phosphor-AKT (p-AKT, Cell Signaling Technology, Danvers, MA, USA), AKT (pan) (total AKT, Cell Signaling Technology, Danvers, MA, USA), heat shock protein 90 (Hsp90, Proteintech, Wuhan, China), tumor susceptibility gene 101 (Tsg101, Proteintech, Wuhan, China), and CD63 (Proteintech, Wuhan, China). Afterwards, the bound antibodies were detected using horseradish peroxidase-conjugated anti-mouse IgG or anti-rabbit IgG (Pierce Chemical, Rockford, IL, USA). Western blot analyses were repeated at least three times to confirm the results.

### 2.5. Lentivirus Vector, Small Interfering RNA (siRNA), and Transfection

EphrinA3 lentiviral vectors and negative control vectors are purchased from GeneChem (Shanghai, China) while siRNA was purchased from GenePharma (Shanghai, China). The HUVECs were seeded in 6-well plates and cultured to reach about 60% cell fusion. After treating with vectors or siRNA and transfection reagents for 6 hours, the mixture was removed and fresh medium was added into the plates. Transfection efficiency was confirmed by using a fluorescence microscope or PCR and Western blot after 72 hours.

### 2.6. Cell Proliferation and Migration Assay

For a cell proliferation assay, HUVECs were seeded in 96-well culture plates and pretreated according to the experiment design. Cell proliferation was detected by CCK-8 (Dojindo, Japan) in accordance with the manufacturer's instructions. For the migration assay, 5 × 10^4^ cells were seeded in 200 *μ*l of serum-free medium in the upper chambers of 24-well plates with inserts (Corning, USA). 500 *μ*l of 5% FBS-ECM was added in the lower chambers. Cells that traversed to the reverse face after 24 h incubation were fixed and stained with crystal violet. Six random fields were photographed with a microscope, and numbers of cells were counted and analyzed by Image-Pro Plus 6.0 (Media Cybernetics, Inc., USA). Migration assays were performed in triplicate.

### 2.7. Tube Formation Assays

After precoating the 48-well plates with Matrigel (BD Biosciences, San Jose, CA, USA) and polymerizing at 37°C for 30 min, 1 × 10^4^ HUVECs were seeded in each well and cultivated with CAL27 CM. Images of the capillary-like structure were photographed with a microscope. The numbers of nodes for each group were counted and analyzed by Image-Pro Plus 6.0 (Media Cybernetics, Inc., USA).

### 2.8. Chromogenic In Situ Hybridization (CISH) and Immunohistochemistry (IHC)

The gene probes targeting miR-210-3p for CISH were purchased from Wuhan Servicebio Technology (Wuhan, China). Tissues of the tumor and normal mucosa were fixed with 4% paraformaldehyde (compounded by diethyl pyrocarbonate water) and embedded in paraffin. After slicing, tissues were deparaffinized in xylene, cleared in an ethanol series, and heat-treated for 15 minutes in antigen repair solution in a microwave oven. For CISH, treat the slices with prehybridization solution and then hybridization solution containing gene probes overnight. Block slices with normal serum after washing, and anti-DIG-AP (anti-digoxin-alkaline phosphatase) was added to them. By developing with BCIP/NBT (Boster, Wuhan, China), the results were observed, recorded, and analyzed. For IHC, treat slices with a CD34 primary antibody (ZSGB-Bio, Beijing, China) at 4°C overnight and then detect them by a peroxidase-conjugated secondary antibody.

### 2.9. Isolation and Analysis of Exosomes

CAL27 cells at 80% confluence were washed three times with PBS and then cultured with FBS-free DMEM. After 24 h, the supernatant was collected and precleared by centrifugation at 800 g for 10 min, 1000 g for 15 min, and 10000 g for 35 min. Exosomes were isolated by ultracentrifugation at 110000 g for 70 min and then washed in PBS under the same condition. Backman (Optima XE-100 Ultracentrifuge) was used to conduct the ultracentrifugation experiments.

Exosomes were observed by transmission electron microscopy HT7700 (HITACHI, Japan), and the hydrodynamic diameter of exosomes was measured with Nano-ZS ZEN 3600 (Malvern Instruments, UK).

### 2.10. Statistical Analysis

The Shapiro-Wilk test was used to assess the normal distribution of the data. Normally distributed data were analyzed with Student's *t*-test and presented as mean ± standard deviation (SD). Categorical variables were expressed as percentage and analyzed with Fisher's exact test. Univariate and multivariate regression models were used to evaluate the association between ephrinA3 expression and clinicopathological variables. Parameters considered statistically significant (*P* < 0.10) in the univariate model were analyzed in the multivariate models. All two-sided *P* values < 0.05 were considered significant. All analyses were carried out using SPSS software (SPSS V.17.0, Chicago, Illinois, USA).

## 3. Results and Discussion

### 3.1. miR-210-3p Expression Was Elevated in OSCC Tissues and Correlated with MVD and Tumor Grade

**Figure 1:**
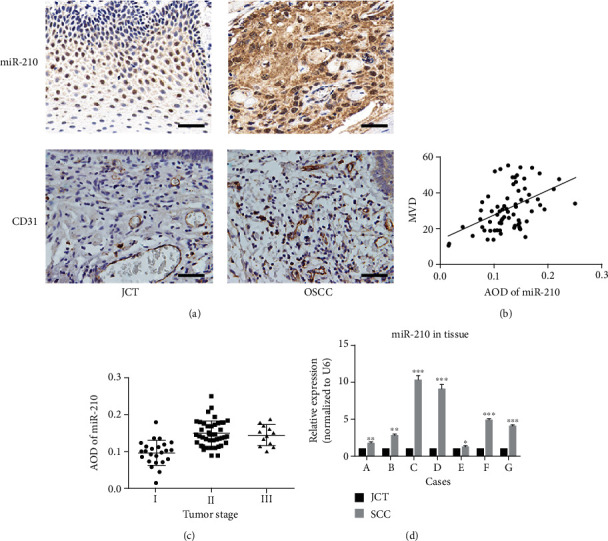
miR-210 expression in tissue. (a) CISH and IHC, respectively, showed miR-210 and CD31 expression in a paraffin section of tissue. Scale bar, 50 *μ*m. (b) Linear regression analysis demonstrated that AOD of miR-210 has a positive correlation with the number of MVD. *R*^2^ = 0.2420, *P* < 0.01. (c) Relationship between the tumor stage and miR-210 expression. (d) RT-PCR showed miR-210 level in fresh OSCC tissue and JCT. ^∗^*P* < 0.05, ^∗∗^*P* < 0.01, and ^∗∗∗^*P* < 0.001.

RNA was extracted from fresh OSCC tissues and a juxta cancerous tissue (JCT). Then, miR-210-3p expression was detected in each sample. miR-210-3p and MVD in a tissue paraffin section indicated that the average optical density (AOD) of miR-210-3p had a positive correlation with MVD (Figures 1(a) and 1(b); *R*^2^ = 0.2420, *P* < 0.01) and miR-210-3p and tumor angiogenesis were associated. The tumor samples were grouped by grade, and a significant difference was observed between grades I and II and between grades I and III (*P* < 0.0001). No statistical difference was observed between grades II and III (Figure 1(c); *P* = 0.0002). miR-210-3p expression was significantly higher in the tumor tissues than in the JCT (Figure 1(d)).

### 3.2. Conditioned Medium (CM) of CAL27 Promoted the Migration and Proliferation of HUVECs and Tube Formation by Upregulating miR-210-3p

**Figure 2:**
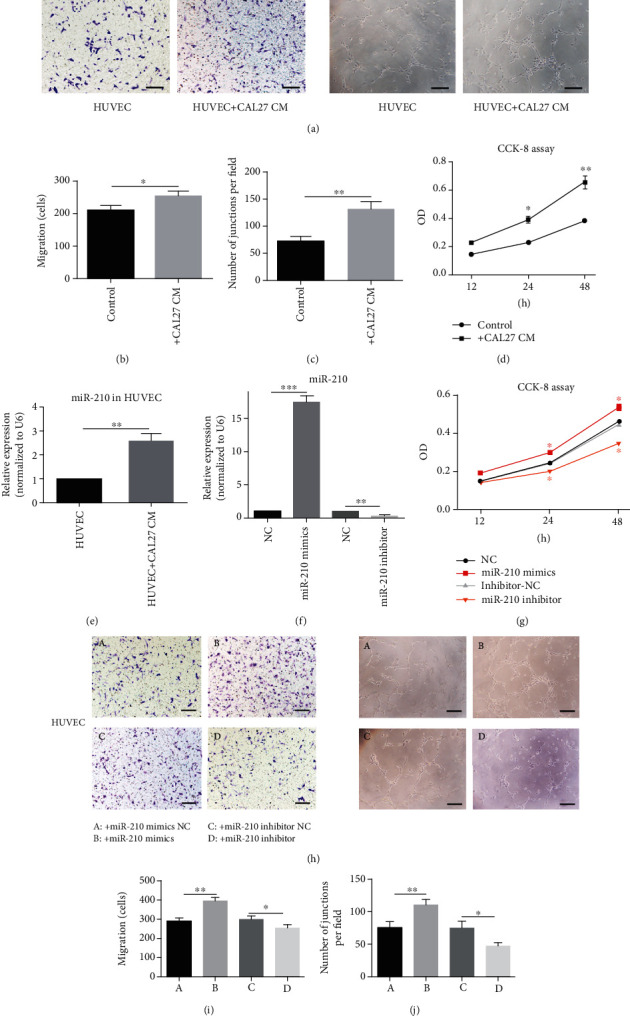
CAL27 CM promotes angiogenesis of HUVEC by elevating miR-210 expression. (a–c) Migration and tube formation assays showed that CAL27 CM could promote migration and tube formation ability of HUVECs. ^∗^*P* < 0.05, ^∗∗^*P* < 0.01. (d) The CCK-8 assay showed that CAL27 CM could promote HUVEC proliferation. *P* < 0.01. (e) RT-PCR showed that the expression of miR-210 in HUVECs was elevated after being treated with CAL27 CM. *P* < 0.01. (f) By being treated with miR-210 mimics, inhibitor, and their negative control (NC) groups, miR-210 was changed accordingly. ^∗∗^*P* < 0.01, ^∗∗∗^*P* < 0.001. (g–j) The CCK-8 assay, migration assay, and tube formation assay showed that compared to NC groups, miR-210 mimics could improve HUVEC angiogenesis while the inhibitor could restrain this capacity. Scale bar, 50 *μ*m. ^∗^*P* < 0.05, ^∗∗^*P* < 0.01.

HUVECs were treated with the CM of CAL27. Then, the CCK-8 assay, Transwell migration assay, and tube formation assay were performed to measure its effect. Transwell migration and tube formation assays showed that the CM of CAL27 promoted the migration HUVECs and tube formation (Figures 2(a)-2(c)). The CCK-8 assay showed that the CM of CAL27 promoted the proliferation of HUVECs (Figure 2(d)). miR-210-3p expression in HUVECs treated with the CM of CAL27 was significantly increased (Figure 2(e)). miR-210-3p participates in tumor development, particularly tumor angiogenesis [33]. To explore whether miR-210-3p plays a role in the proangiogenic progress of the CM of CAL27, we infected HUVECs with miR-210-3p mimics (Figure 2(f)). An increase in miR-210-3p expression enhanced the proliferation and migration of HUVECs and tube formation (Figures 2(g)-2(j)). Inversely, compared with the control group, miR-210-3p expression downregulated by an inhibitor suppressed the proliferation and migration of HUVECs and tube formation (Figures 2(g)-2(j)). Therefore, the CM of CAL27 promoted HUVEC migration, proliferation, and tube formation by upregulating the miR-210-3p expression in HUVECs.

### 3.3. miR-210-3p Decreased the EphrinA3 Protein in HUVECs

**Figure 3:**
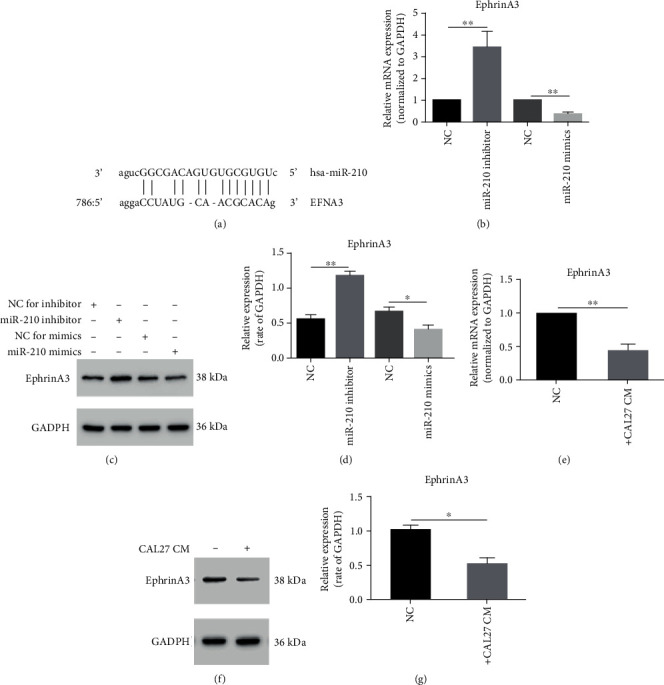
miR-210 targets 3′UTR of EFNA3. (a) Binding site between miR-210 and EFNA3. (b–d) RT-PCR and Western blot showed that ephrinA3 in HUVECs changed when treated with miR-210 mimics, inhibitor, and their NC groups. Mimics of miR-210 could reduce ephrinA3, and the inhibitor could increase its level. ^∗^*P* < 0.05, ^∗∗^*P* < 0.01. (e) After treating with CAL27 CM, RT-PCR showed that mRNA expression of ephrinA3 in HUVECs was downregulated. *P* < 0.01. (f, g) Western blot showed that ephrinA3 protein in HUVECs was also reduced by treating with CAL27 CM. *P* < 0.05.

EFNA3 is a direct target of miR-210-3p at http://microRNA.org/ (Figure 3(a)), and this conclusion has been verified in several articles [17, 22, 34, 35]. Therefore, we used PCR and Western blot to detect ephrinA3 levels in HUVECs treated with miR-210-3p mimics and inhibitors. Compared with the negative control group, overexpressed miR-210-3p significantly reduced ephrinA3 protein expression, whereas miR-210-3p inhibition remarkably downregulated ephrinA3 protein expression (Figures 3(b)-3(d)). The PCR and Western blot results showed that the expression of ephrinA3 protein in HUVECs treated with the CM of CAL27 was downregulated (Figures 3(e)-3(g)).

### 3.4. EphrinA3 Influences the Angiogenic Ability of HUVECs

**Figure 4:**
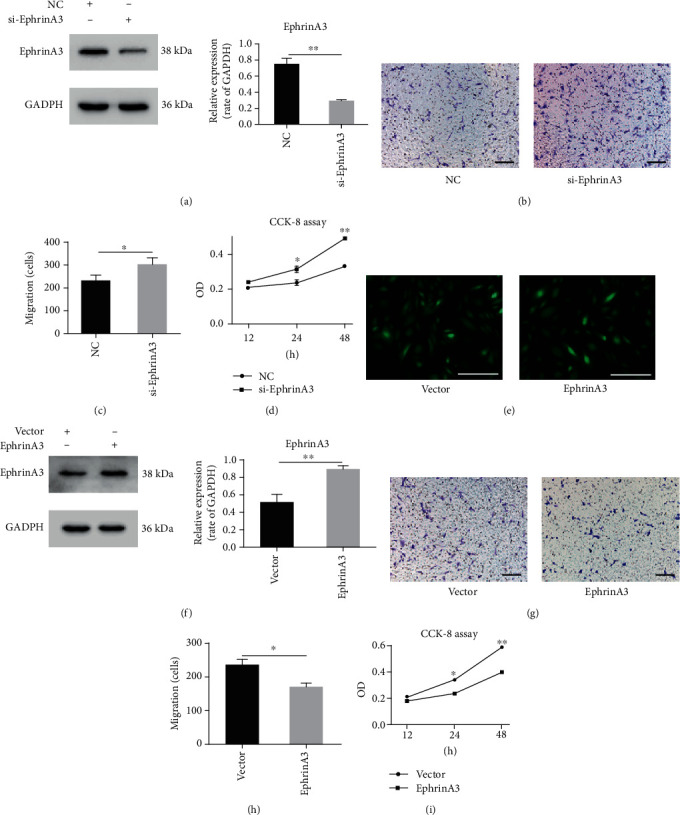
EphrinA3 could influence the migration and proliferation of HUVECs. (a) Western blot showed that EphrinA3 was downregulated by si-EphrinA3 compared to NC. *P* < 0.01. (b–d) Migration and CCK-8 assays showed that migration and proliferation were promoted in HUVECs when ephrinA3 was reduced by si-EphrinA3. (e) Fluorescence microscopy suggested that the transfection of the vector and lentivirus was successful, and its efficiency was about 90%. Scale bar, 50 *μ*m. (f) EphrinA3 was upregulated by lentivirus. *P* < 0.01. (g–i) Migration and proliferation of HUVECs were restrained after upregulating the ephrinA3. ^∗^*P* < 0.05, ^∗∗^*P* < 0.01. Scale bar, 50 *μ*m.

EphrinA3 is involved in angiogenesis [22] [36, 37]. We used siRNA and its negative control to reduce the expression of ephrinA3 in HUVECs. Western blot results showed that siRNA downregulated the level of ephrinA3 in HUVECs (Figure 4(a)). CCK-8 and Transwell migration assays were applied to investigate the influence of ephrinA3 on HUVECs. Compared with the negative control group, HUVECs infected with siRNA showed a strong ability to migrate and proliferate (Figures 4(b)-4(d)). Then, we constructed lentiviral vectors containing green fluorescence protein genes to overexpress ephrinA3 in HUVECs. The transfection efficiency of ephrinA3 lentiviral and negative control vectors was almost 90% when observed under a fluorescence microscope after transfection (Figure 4(e)). The Western blot results verified that ephrinA3 was upregulated (Figure 4(f)). Then, CCK-8 and Transwell migration assays were conducted to confirm whether ephrinA3 overexpression slows down the proliferation and inhibits the migration of HUVECs (Figures 4(g)-4(i)).

### 3.5. EphrinA3 Influences the Proliferation and Migration of HUVECs by Activating the PI3K/AKT Pathway

**Figure 5:**
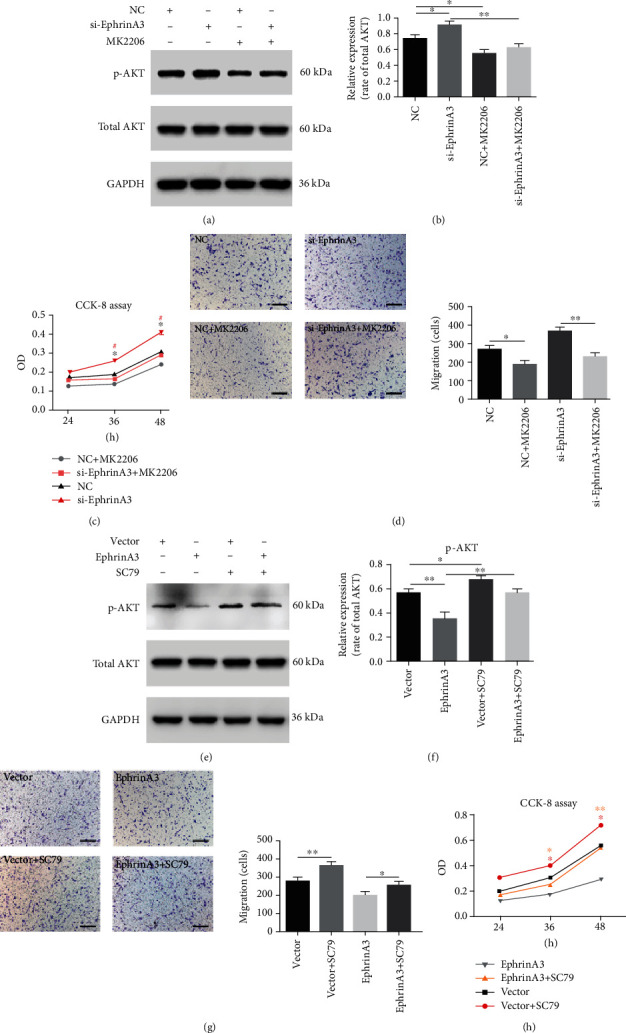
EphrinA3 regulated migration and proliferation in HUVECs by activating the PI3K/AKT pathway. (a, b) Western blot showed that after transfection with si-EphrinA3, the p-AKT/total AKT rate in HUVECs was increased. MK2206 could reduce the phosphorylation of AKT. (c, d) The promotion of HUVEC migration and proliferation by downregulation of ephrinA3 was blocked by MK2206. (e, f) The p-AKT/total AKT rate was declined when ephrinA3 was upregulated by lentivirus. SC79 could promote the phosphorylation of AKT. (g, h) The suppression on HUVEC migration and proliferation as a result of the upregulation of ephrinA3 was inhibited by SC79.

Eph/ephrin can influence a variety of pathways, such as the PI3K/AKT, ERK, Wnt, and RhoA/ROCK pathways [38–40]. For the identification of the specific pathways involved in the process of ephrinA3 influencing HUVECs, the targets of potential pathways were detected in the cells by Western blot, and the results showed that the p-AKT/AKT level increased after ephrinA3 expression was downregulated, indicating the activation of the PI3K/AKT pathway (Figure 5(a)). The AKT pathway participates in the normal physiological processes of HUVECs, including proliferation and migration, and in the pathological course by cancer cells [41, 42]. Then, whether changes in the AKT pathway influence the proliferation and migration of HUVECs was investigated by using the AKT inhibitor MK2206 and agonist SC79. After HUVECs were treated with MK2206, the p-AKT/AKT rate and cancer cell proliferation and migration were reduced (Figures 5(b)-5(d)). By contrast, SC79 enhanced the proliferation and migration of the HUVECs by upregulating the p-AKT/AKT rate (Figures 5(e)-5(h)). On this basis, ephrinA3 regulated the migration and proliferation of HUVECs by phosphorylating AKT, that is, the activation of the PI3K/AKT pathway.

### 3.6. CAL27 Delivers miR-210-3p to HUVECs by Exosomes

**Figure 6:**
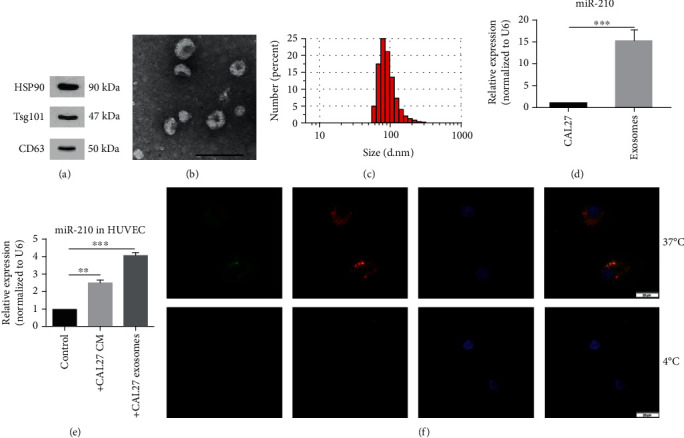
CAL27 delivered miR-210 into HUVECs by secreting exosomes. (a) An exosomal marker was detected by Western blot in exosomes isolated from the CAL27 supernatant. (b, c) Exosomes released by CAL27 were identified using transmission electron microscopy and dynamic light scattering analysis. Scale bar, 200 nm. (d) miR-210 was identified to exist in the exosomes. *P* < 0.001. (e) miR-210 in HUVECs was upregulated after stimulation with CAL27 exosomes. ^∗∗^*P* < 0.01, ^∗∗∗^*P* < 0.001. (f) Images under a fluorescence microscope showed that miR-210 can be packaged into exosomes, and then, at 37°C, exosomes could be uptaken into HUVECs. At 4°C, exosomes could not be absorbed into cells so that there was no fluorescent light in HUVECs. Scale bar, 20 *μ*m.

We speculated the reason that the CM of CAL27 upregulates the expression of miR-210-3p in HUVECs; that is, exosomes might be a tool for CAL27 to deliver miR-210-3p to HUVECs. The protein biomarkers of exosomes, namely, HSP90, Tsg101, and CD63, were detected by Western blot (Figure 6(a)). The exosomes isolated from the culture supernatant of CAL27 were identified by transmission electron microscopy and dynamic light scattering analysis (Figures 6(b) and 6(c)). In addition, exosomal miR-210-3p levels were higher in the exosomes isolated from CAL27 cells than in those in CAL27 cells (Figures 6(d) and 6(e)). Isolated exosomes from CAL27 transfected with the fluorescein isothiocyanate- (FITC-) tagged miR-210-3p were labelled with the fluorescent dye PKH26. HUVECs were treated with exosomes as described above. Then, FITC and PKH26 fluorescence was observed within HUVECs under the fluorescence microscope, and no FITC or PKH26 fluorescence was observed in the control group cultured at 4°C (Figure 6(f)).

These results indicated that CAL27 secreted exosomal miR-210-3p that can be delivered into HUVECs.

## 4. Conclusions

Extensive literature indicated a significant and close relationship between microRNAs and tumor development involving angiogenesis, metastasis, and tumor immunity. miR-210-3p presents its carcinogenic features. As a potential diagnostic biomarker, miR-210-3p in the sera of patients with Graves' disease and colorectal cancer has remarkably higher levels than that in the sera of the healthy control groups [43, 44]. The expression of miR-210-3p in the sera of pregnant women with preeclampsia was upregulated; hence, some relationship may exist between miR-210-3p and preeclampsia pathogenesis [45]. OSCC tissues express more miR-210-3p than normal oral mucosa, and this finding is consistent with our results observed in OSCC tissues [46, 47]. Cellular viability, colony formation, S phase percentage, and invasiveness of malignant peripheral nerve sheath tumor (MPNST) cells were increased by overexpressing miR-210, whereas the suppression of miR-210 expression resulted in the inhibition of the proliferation and invasion of MPNST cells [35].

Inevitably, tumor growth is accompanied by hypoxia, which can promote tumor angiogenesis and invasion [48–50]. In addition to the effect of miR-210-3p on angiogenesis, hypoxia can increase the expression of miR-210-3p by inducing the demethylation of the promoter and tumor proliferation and autophagy in schwannoma cells [33]. miR-210-3p is associated with the elevation of the expression of local VEGF as a key factor in promoting angiogenesis and neurogenesis [51]. However, whether miR-210-3p promotes angiogenesis in OSCC remains unclear. Although miR-210-3p influences tube formation in endothelial cells by targeting ephrinA3, mechanism of this phenomenon is still unknown [17, 22].

Several papers have reported that EFNA3 is the target gene of miR-210. Therefore, we did not repeat the dual-luciferase reporter assay in our research. However, we did confirm that miR-210 regulates ephrinA3. On the biological effect of ephrinA3 in oncogenesis, knocking out EFNA3 can promote the cellular viability and invasiveness of MPNST cells; hence, EFNA3 may serve as a tumor suppressor [52]. The effect of miR-210 on HUVECs is complicated because of the variety of targeting genes of microRNAs, and many genes apart from EFNA3 may participate in the process. Thus, further experiments will be designed to explain the action mechanism of miR-210 delivered by OSCC exosomes.

Tumor cells have rapid growth rates and thus require more oxygen and nutrients than normal cells, but the blood vessels of adults remain stationary and hardly form new branches under normal physiological conditions [53]. In the initial stage of growth, the tumor has no relationship with the vascular system. Therefore, the vascular system cannot be activated until vessel growth occurs in the surrounding capillaries under the stimulation of the tumor itself [54]. Thus, tumor cells secrete growth factors, such as VEGF, and many other substances, such as microvesicles and exosomes, to change the states of vascular endothelial cells in the TME by improving the ability of proliferation, migration, and tubule formation during tumor development. Further exploration about the molecular mechanism of tumor angiogenesis will provide a possibility of improving the existing methods for antitumor treatment. In the present research, OSCC tissues had higher miR-210-3p expression than JCT, and the expression of miR-210-3p had a positive correlation with tumor MVD. Hence, miR-210-3p may participate in tumor angiogenesis, and it played some roles in tumorigenesis. The statistical differences of miR-210-3p level can be observed between grades I and II and between I and III but not between II and III. The reason might be that miR-210-3p played vital roles in the preliminary period of tumor development and the early stage when the tumor established vascular connections with the microenvironment. When the tumor develops to some stage, some molecules might affect the tumor angiogenesis. However, the deviation due to sample size cannot be ignored. Therefore, we will increase our sample capacity to obtain precise results in our subsequent research.

The roles of exosomes released by tumor cells in the TME cannot be disregarded, and abundant contents made exosomes to be the information media between cells. Exosomes can transfer tumorigenic and angiogenic factors from tumor cells to surrounding cells in the TME to induce the activation of tumor angiogenesis and promote the formation of premetastatic niche [55]. Exosomes from head and neck squamous cell carcinoma cell lines facilitate angiogenesis by reprogramming and modulating endothelial cells [56]. Various bioactive compounds can be enveloped into exosomes. MicroRNAs have been widely studied. miR-210-3p is present in exosomes in many cell lines [57, 58]. In the present study, miR-210-3p was detected in exosomes isolated from the CM of CAL27. Exosomes isolated from the CM of CAL27 delivered miR-210-3p to recipient cells, at least partly inducing the upregulation of miR-210-3p in HUVECs. By elevating miR-210-3p expression level in HUVECs, CAL27 regulated angiogenesis in the cells. In addition, we added the knowledge about the miR-210-3p-ephrinA3 axis with studies in HUVECs. miR-210-3p targeted and downregulated the expression of ephrinA3, possibly increasing the phosphorylation rate of AKT in the PI3K/AKT signaling pathway to enhance the proliferation and migration of HUVECs. Given that miR-210-3p expression can be enhanced by the CM and exosomes of CAL27 through other means, except delivery, the mechanisms involved in the modulation of AKT phosphorylation by ephrinA3 should be further explored and verified.

## Figures and Tables

**Table 1:** 

Primer and direction	Sequence
miR-210-3p forward	5′-CTGTGCGTGTGACAGCGG-3′
U6 forward	5′-ATGGACTATCATATGCTTACCGTA-3′
GAPDH forward	5′-GGAGCGAGATCCCTCCAAAAT-3′
GAPDH reverse	5′-GGCTGTTGTCATACTTCTCATGG-3′
EphrinA3 forward	5′-TACTACTACATCTCCACGCCCACTC-3′
EphrinA3 reverse	5′-TCCCGCTGATGCTCTTCTCAA-3′

## Data Availability

All data generated or analyzed during this study are included in this article.
